# The effects of transdermal and oral oestrogen replacement therapy on colorectal cancer risk in postmenopausal women

**DOI:** 10.1038/sj.bjc.6601438

**Published:** 2004-01-06

**Authors:** I Csizmadi, J-P Collet, A Benedetti, J-F Boivin, J A Hanley

**Affiliations:** 1 Centre for Clinical Epidemiology, S.M.B.D. Jewish General Hospital, 3755 Chemin de la Côte Ste-Catherine, Montreal, Quebec, Canada H3T 1E2; 2 Department of Epidemiology and Biostatistics, McGill University, 1020 Pine Avenue West, Montreal, Quebec, Canada H3A 1A2

**Keywords:** transdermal oestrogen, oral oestrogen, colorectal cancer, postmenopausal, case-control study

## Abstract

The aim of this study was to examine the effects of oral and transdermal oestrogen replacement therapy on the risk of colorectal cancer. Data from a nested case–control study, designed to investigate the effect of hormone replacement therapy (HRT) on colorectal cancer were analysed. New cases of colorectal cancer, diagnosed between 1992 and mid-1998 (*N*=1197), were identified using the Saskatchewan Cancer Agency cancer registry. Women ⩾50 years of age, eligible for coverage by the Saskatchewan Prescription Drug Plan, were included in the study. Four controls per case were age matched to cases, using incidence density sampling. The outpatient prescription drug plan database was used to ascertain oestrogen prescriptions. Women were classified according to history of transdermal (TDE) and oral (OE) oestrogen use. Conditional logistic regression was used to estimate odds ratios (ORs) and 95% confidence intervals (CIs). Compared with women who had never used HRT, ORs for <3 and ⩾3 years of TDE use and colorectal cancer were 0.69 (95% CI: 0.43–1.10) and 0.33 (95% CI: 0.12–0.95), and for OE use were 0.90 (95% CI: 0.73–1.01) and 0.75 (95% CI: 0.60–0.93), respectively. The risk reduction for colorectal cancer with TDE may be greater in magnitude than that which has been reported for oral HRT.

The release of results from the Women's Health Initiative (WHI) (Writing Group for the Women's Health Initiative Investigators, 2002) and the Heart and Oestrogen/Progestin Replacement Study (HERS II) (Grady *et al*, 2002; Hulley *et al*, 2002) trials in July 2002 clarified some of the risks and benefits of hormone replacement therapy (HRT) that have been debated for decades. Currently, most experts appear to agree that only women experiencing menopausal symptoms should be prescribed oral combined oestrogen–progestagen therapy (Blake *et al*, 2002), since among older postmenopausal women the evidence suggests that the risk of overall harm outweighs the potential for benefit. Experts also agree that systemic hormone therapy is the most effective treatment for vasomotor and other menopausal symptoms (Greendale *et al*, 1998; Blake *et al*, 2002). Hence, for 10–25% of postmenopausal women who experience distressing menopausal symptoms, HRT will continue to be a therapy of choice (Yusuf and Anand, 2002).

The results from the WHI (Writing Group for the Women's Health Initiative Investigators, 2002) randomised controlled trial and numerous observational studies are consistent in their findings that suggest that HRT is protective against colorectal cancer (Furner *et al*, 1989; Jacobs *et al*, 1994; Folsom *et al*, 1995; Newcomb and Storer, 1995; Persson *et al*, 1996; Kampman *et al*, 1997; Fernandez *et al*, 1998; Grodstein *et al*, 1998; Prihartono *et al*, 2000). All of these studies, however, have focused exclusively on the use of oral oestrogen (OE). This may be due in part to the higher prevalence of OE use among women, compared with transdermal oestrogen (TDE). However, since the availability of TDE in the mid-1980s, its use has been increasing in both Canada and the US (Wysowski *et al*, 1995).

While evidence from clinical studies demonstrate important metabolic and biologic differences between oral and transdermal HRT (Chetkowski *et al*, 1986; IARC Working Group on Hormonal Contraception and Post-menopausal Hormonal Therapy, 1999; Gruber and Huber, 2001; Campagnoli *et al*, 2002), limited information is available relating the mode of hormone delivery (oral *vs* transdermal) to clinically important outcomes. Faced with the decision to begin HRT or not, women need to weigh the health risks against the benefits of menopausal symptom control. Provided that there is sufficient knowledge about the health effects of various HRT formulations and modes of delivery, women and their physicians could potentially individualise treatment with HRT to minimise the health risks and optimise benefit.

In this population-based case–control study, we examine the effects of transdermal and OE administration on the risk of colorectal cancer in peri- and postmenopausal women.

## MATERIALS AND METHODS

### Study design

A subset of data from a large population-based nested case–control study were analysed to investigate the effects of oral and TDE on the risk of colorectal cancer risk among peri- and postmenopausal women in Saskatchewan, a province in Canada. Saskatchewan Health (SH) is a provincial government department that maintains electronic health service databases as part of its publicly funded health system. These databases were used as sources of health services information for this study. Over 99% of the Saskatchewan population are registered with Saskatchewan Health and about 91% are eligible for outpatient prescription drug benefits (Downey *et al*, 2000). The majority of those not eligible are registered members of First Nations who receive prescription drug benefits under a federal program.

### Study subjects

Women, 50 years of age and older, diagnosed with histologically confirmed colorectal cancer between January 1, 1981 and June 30, 1998 were identified using records from the Saskatchewan Cancer Agency (SCA) cancer registry, which has been in existence since 1932 and computerised since 1970 (Rawson and Robson, 2000). Cancer cases are identified by the SCA using both pathology reports and physician service claims, and therefore the registry is one of the most complete in Canada.

All cases and controls eligible for this study had to have had at least 5 years of registration with SH, had to have been eligible for outpatient prescription drug benefits and not have been diagnosed with cancer (except for nonmelanoma skin cancer and cancer of the cervix *in situ*) prior to the index date. Saskatchewan Cancer Agency records were used to verify that participants had not had previous cancer diagnoses.

Using SH electronic records, four controls were matched on the year of birth (±1 year) to each identified case. Specifically, a control had to have been living in Saskatchewan at the time the colorectal cancer case (to whom she was matched) was diagnosed with cancer (index date). For each case, 16 potential controls were randomly sampled using incidence density sampling (Rothman and Greenland, 1998), with replacement, from the pool of eligible controls. These controls were screened for the absence of a history of diagnosed cancer prior to a specific index date, using records of the SCA. Women identified as having had cancer prior to their index dates were excluded as potential controls. Controls who did not have histories of cancer prior to their index dates were retained and, from them, four controls per case were randomly sampled without replacement. In this analysis, we included only women with index dates (for each case–control set, the date of the case cancer diagnosis) on January 1, 1992 or later.

### Ascertainment of oestrogen exposure

Dispensing data were ascertained for all forms of oestrogen: oral, transdermal patch, and vaginal creams and rings listed in the Saskatchewan Formulary. For each woman in the study, the type of formulation, strength, and number of oestrogen units dispensed, prior to index dates, were obtained from the records of the outpatient prescription drug plan database, originally established in 1975. Prescription drug dispensing data were ascertained for each woman from the time her prescription drug coverage began, until the date of colorectal cancer diagnosis for cases or assigned index dates for controls.

Transdermal oestrogen replacement was listed in the Formulary on July 1, 1988 with unrestricted coverage, and it was moved to restricted coverage on January 1, 1997 (oestrogen exposure after June 30, 1996 is not relevant for the current analysis, see *Definitions of oestrogen exposure*). There was also a break in complete data capture for all dispensed prescriptions between July 1, 1987 and December 31, 1988. For this period of time, the HRT exposure assigned to each woman was based on her exposure status before and after the 18-month period. No exposure was assigned if she did not have a prescription dispensed during the year before or after the break, one additional year of transdermal or OE exposure was assigned if she had had at least one transdermal or OE prescription dispensed during the year prior to, or, during the year following the break.

### Definitions of oestrogen exposure

Women were classified according to history of HRT use: TDE use only; OE use only; or use of TDE or OE without exclusion of women who may have used both OE and TDE. Exposure to HRT during the 2 years prior to index dates was not included in exposure calculations, because of evidence suggesting that women who begin to feel unwell as a result of undiagnosed cancer may discontinue HRT, leaving primarily healthy women as HRT users (Sturgeon *et al*, 1995). In addition, exposure in the more distant past is likely to be more relevant for cancer incidence. A woman was identified as an ‘ever’ user if she had received at least one prescription of TDE or OE prior to her index date, excluding the 2 years immediately preceding it. This reference point will hereafter be referred to as the reference date. Women who had used HRT only during the 2 years prior to their index date and women who had received prescriptions for oestrogen containing vaginal creams or rings only, were included in the reference groups. Vaginal oestrogen use has been associated with poor compliance, and has been documented to have varying degrees of absorption with primarily local rather than systemic effects (Santen *et al*, 2002).

A woman was classified as having been exposed during any given year if she had been dispensed at least one prescription of OE or TDE oestrogen in that year. The cumulative number of years of exposure was determined and duration of use was classified according to short-term (<3 years) or long-term use (⩾3 years). A 3-year threshold was selected, *a priori*, since it was felt that too few women would have had the opportunity to have been exposed to TDE for a longer period of time.

### Ascertainment of covariates

The use of oral contraceptives, cardiovascular system (CVS) drugs, central nervous system (CNS) drugs, and prescription nonsteroidal anti-inflammatory drugs (NSAIDs) and vitamins, and other hormones were also ascertained from the outpatient prescription drug plan database for the period of time preceding index dates. The history of having had a sigmoidoscopy and the frequency of physician visits during the 5-year period prior to index dates were ascertained for all cases and controls, using records of the medical services database.

### Statistical analysis

Conditional logistic regression was used to calculate the odds ratios (ORs) and 95% confidence intervals (CIs). The effect of age was controlled for with matching and, where matching was broken, age-adjusted ORs were obtained with the inclusion of age in the regression model. The confounding effects of covariates, identified *a priori*, were assessed using the criteria of observing a change in ORs of 10% or more. Backward and forward model selection methods were used to identify the best regression model.

All associations between TDE and OE and risk of colorectal cancer were examined among all women and for the subgroup who had not had a sigmoidoscopy 3–5 years prior to index dates. The analysis could not be carried out among women who had had a sigmoidoscopy during this period of time, because too few women had had the procedure.

An unmatched analysis was also carried out where a direct comparison was made between TDE and OE on the risk of colorectal cancer. In this analysis, women exposed to TDE were compared with women who had used OE exclusively (reference group). Age-adjusted ORs and 95% CIs were calculated using unconditional logistic regression.

A statistical test for linear trend was carried out for the duration of HRT use by redefining short and longer duration (<3 or ⩾3 years) of TDE and OE use as ordinal variables, and assessing their level of significance in a logistic regression model. All statistical analyses were carried out using SAS software (SAS Institute, Version 8, 1999).

## RESULTS

In all, 1261 cases and 4916 age-matched controls had index dates between January 1, 1992 and mid-1998. Of these, 1197 cases had histologically confirmed colorectal cancer. The health-related characteristics of these 1197 colorectal cancer cases and their 4669 controls are outlined in Table 1

**Table 1:** Health-related characteristics of cases and controls

	**Cases (*n*=1197) (%)**	**Controls (*n*=4669) (%)**
Age in years at index date (mean (SD))	73.8 (10.2)	73.8 (10.2)
		
*Ever use of prescription drugs prior to index date*		
Vaginal oestrogen cream or ring only	14.5	14.2
Oral oestrogen	22.8	25.3
Transdermal oestrogen	2.7	4.1
Cardiovascular drugs	68.0	66.0
Central nervous system drugs	70.8	73.1
Oral contraceptives	1.4	1.4
Other hormones and substitutes	37.7	40.0
Vitamins	18.4	18.2

*NSAIDs use in years before the index date*		
1–5	49.1	56.5
6–10	52.2	57.4
11–15	56.0	58.7

*History of sigmoidoscopy or colonoscopy*		
Year before the index	70.0	2.3
2–5 years before the index	9.5	8.5
3–5 years before the index	6.5	6.9

*Frequency of doctor visits in year before index date*		
0–2	3.1	20.1
3–7	6.5	26.2
8–14	25.3	25.0
15+	65.1	28.8

*Frequency of doctor visits 2-5 years before index date*		
0–9	12.9	13.8
10–24	20.5	20.3
25–59	39.0	42.2
60+	27.5	23.7

. Cases and controls had mean ages of 73.8 (s.d. 10.2) years. Just over 14% of women had received at least one prescription of vaginal oestrogen creams or rings, without having been dispensed OE or TDE. In total, 22.8 and 2.7% of cases had been dispensed OE and TDE, respectively, compared with 25.3 and 4.1% of controls. The use of prescription vitamin, cardiovascular, and CNS drugs did not differ between cases and controls, but there was a tendency for controls to use more NSAIDs during the 15 years prior to index dates. Not surprisingly, important differences were observed between cases and controls for having had a sigmoidoscopy during the year immediately preceding the index date (70.0 *vs* 2.3% for cases and controls, respectively). Only minor differences were observed for having had a sigmoidoscopy during the 2–5 and 3–5 years preceding the index dates. During the year preceding the index date, 65.1% of the cases also visited their physician 15 times or more, compared with 28.8% of controls. The frequency of physician visits 2–5 years prior to index dates did not differ greatly between cases and controls.

The number of women, among cases and controls, exposed to various oestrogen formulations prior to the reference date, are presented in Table 2

**Table 2:** Use of oral (OE), transdermal (TDE), and vaginal oestrogen creams or rings among cases and controls prior to the reference date (2 years prior to index date)

	**Cases (*N*=1197)**		**Controls (*N*=4669)**	
**Formulation**	***N***	**%**	***N***	**%**
Never use of HRT or vaginal creams or rings	764	63.8	2838	63.5
Vaginal creams or rings only	177	14.8	661	14.2
OE only^a^	230	19.2	1013	21.7
OE (may have used TDE)^b^	251	21.0	1112	23.8
TDE only^c^	5	<0.1	58	1.3
TDE (may have used oral)^d^	26	2.2	157	3.4

a In total, 62 (27%) of these cases and 340 (34%) of these controls had also been dispensed at least one prescription of vaginal cream or ring prior to the reference date.

b A total of 66 (26%) of these cases and 376 (34%) of these controls had also been dispensed at least one prescription of vaginal cream or ring prior to the reference date.

c One (20%) of these cases and 16 (28%) of these controls had also been dispensed a prescription of vaginal cream or ring prior to the reference date.

d Five (19%) of these cases and 52 (33%) of these controls had also been dispensed a prescription of vaginal cream or ring prior to the reference date.

. A total of 63.8% of cases and 63.5% of controls had never been exposed to any oestrogen formulation. Except in the case of vaginal oestrogen, where an equal percentage of women had been dispensed prescriptions, slightly more controls than cases had been dispensed at least one prescription of OE or TDE. Some of the women had received OE and TDE exclusively, while others had received other oestrogen formulations at some time.

Prescription drug use only minimally altered ORs for oestrogen and colorectal cancer (less than 10%), and therefore only age-adjusted ORs are presented. ‘Ever’ use of OE was associated with an OR of 0.82 (95% CI: 0.70–0.97) (Table 3

**Table 3:** Age-adjusted odds ratios and 95% confidence intervals for incidence of colorectal cancer with ever use of oral and transdermal oestrogen

	**All women**				**Among women who had not had a sigmoidoscopy^a^**			
	**Cases (*N*=1197)**	**Controls (*N*=4669)**	**OR^b^**	**95% CI**	**Cases (*N*=1115)**	**Controls (*N*=4366)**	**OR^b^**	**95% CI**
Unexposed^c^	941	3499	1.00		880	3297	1.00	
Oral^d^	251	1112	0.82	0.70–0.97	231	1015	0.85	0.72–1.01
Transdermal^d^	26	157	0.60	0.39–0.92	23	144	0.58	0.37–0.92
Transdermal only	5	58	0.32	0.13–0.79	4	54	0.27	0.10–0.76

a Sigmoidoscopy 3–5 years prior to the index date.

b Cases and controls matched on age.

c Includes women who may have used vaginal oestrogen creams or rings only and women who had received an HRT prescription during the 2-year period prior to the index date.

d May have used other HRTs.

). The ‘ever’ use of TDE was associated with an OR of 0.60 (95% CI: 0.39–0.92). Among women who had ‘ever’ used only TDE, the OR associated with colorectal cancer was 0.32 (95% CI: 0.13–0.79). The ORs for ‘ever’ use of OE or TDE among women who had not had sigmoidoscopies were not markedly different from that of the entire group.

The association between colorectal cancer risk and duration of OE use is outlined in Table 4

**Table 4:** Age-adjusted odds ratios and 95% confidence intervals for incidence of colorectal cancer and duration of oral oestrogen use

	**All women**					**Among women who had not had a sigmoidoscopy^a^**			
	**Cases (*N*=1197)**	**Controls (*N*=4669)**	**OR^b^**	**95% CI**	***P* for trend**	**Cases (*N*=1115)**	**Controls (*N*=4366)**	**OR^b^**	**95% CI**
*Oral oestrogen*^c^									
Unexposed^d^	941	3499	1.00			880	3297	1.00	
<3 years	135	548	0.90	0.73–1.01		126	504	0.93	0.75–1.16
⩾3 years	116	564	0.75	0.60–0.93	0.01	105	511	0.77	0.61–0.97

*Oral oestrogen only*									
Unexposed^d^	941	3499	1.00			880	3297	1.00	
<3 years	124	511	0.89	0.72–1.10		116	472	0.92	0.74–1.15
⩾3 years	106	502	0.77	0.61–0.96	0.02	96	453	0.79	0.62–1.01

a Sigmoidoscopy 3–5 years prior to the index date.

b Cases and controls matched on age.

c May have used transdermal oestrogen.

d Includes women who may have used vaginal oestrogen creams or rings only and women who had received an HRT prescription during the 2-year period prior to the index date.

. Odds ratios for short-term, less than 3 years of use (OR=0.90 (95% CI: 0.73–1.01)), and longer duration of 3 years of OE use or more (0.75 (95% CI: 0.60–0.93)), were below one for OE users who may have also used TDE oestrogen. This was also true for women who had used OE only (OR=0.89 (95% CI: 0.72–1.10) and 0.77 (95% CI: 0.61–0.96), for short- and long-term use, respectively). The statistical tests for linear trend for duration of use were statistically significant for both OE and OE only (*P*=0.01 and 0.02, respectively). Similar point estimates were obtained for women who had not had a sigmoidoscopy 3–5 years prior to index dates. The test for linear trend remained significant for all OE use (*P*=0.02), but not for OE only use (*P*=0.07).

For TDE use, among women of whom some had used OE in the past, the ORs were 0.69 (95% CI: 0.43–1.10) for less than 3 years of TDE use, and 0.33 (95% CI: 0.12–0.95) for 3 or more years of TDE use (Table 5

**Table 5:** Age-adjusted odds ratios and 95% confidence intervals for incidence of colorectal cancer and duration of transdermal oestrogen use

	**All women**					**Among women who had not had a sigmoidoscopy^a^**			
	**Cases (*N*=1197)**	**Controls (*N*=4669)**	**OR^b^**	**95% CI**	***P* for trend**	**Cases (*N*=1115)**	**Controls (*N*=4366)**	**OR^b^**	**95% CI**
Transdermal oestrogen^c^										
Unexposed^d^	941	3499	1.00			880	3297	1.00		
<3 years	22	115	0.69	0.43–1.10		20	106	0.68	0.42–1.11	
⩾3 years	4	42	0.33	0.12–0.95	0.01	3	38	0.29	0.09–0.94	

a Sigmoidoscopy 3–5 years prior to the index date.

b Cases and controls matched on age.

c May have used oral oestrogen.

d Includes women who may have used vaginal oestrogen creams or rings only and women who had received an HRT prescription during the 2-year period prior to the index date.

). The linear test for trend was statistically significant (*P* for trend=0.01). Similar results were observed for TDE use among women who had not had a sigmoidoscopy 3–5 years prior to index dates, with the test for linear trend remaining significant (*P*=0.01). For TDE users only, the same trend in decreasing ORs was observed with increasing duration of use; however, only five cases had been exposed to TDE only and only one case was exposed for 3 or more years.

An analysis was carried out to determine the association between TDE use and colorectal cancer risk among HRT users (cases *N*=235; controls *N*=1071) (Table 6

**Table 6:** Age-adjusted odds ratios and 95% confidence intervals for incidence of colorectal cancer associated with duration of transdermal oestrogen use among postmenopausal oestrogen users (oral oestrogen users are the reference group)

	**All women**				**Among women who had not had a sigmoidoscopy^a^**			
	**Cases (*N*=235)**	**Controls (*N*=1071)**	**Age-adjusted OR**	**95% CI**	**Cases (*N*=216)**	**Controls (*N*=979)**	**Age-adjusted OR**	**95% CI**
OE use only	230	1013	1.00		212	925	1.00	
TDE use only	5	58	0.37	0.15–0.94	4	54	0.32	0.12–0.90

a Sigmoidoscopy 3–5 years prior to the index date.

). The reference category in this analysis comprised women who had used OE exclusively. Relative to these, women who had used TDE only had an OR of 0.37 (95% CI: 0.15–0.94) and, similarly, among the women who had not had a sigmoidoscopy 3–5 years prior to the index dates, the OR was 0.32 (95% CI: 0.12–0.90).

## DISCUSSION

The majority of observational studies (Furner *et al*, 1989; Jacobs *et al*, 1994; Folsom *et al*, 1995; Newcomb and Storer, 1995; Persson *et al*, 1996; Kampman *et al*, 1997; Fernandez *et al*, 1998; Grodstein *et al*, 1998, 1999; Nanda *et al*, 1999; Prihartono *et al*, 2000) and the results from the WHI (Writing Group for the Women's Health Initiative Investigators, 2002) randomised controlled trial suggest that oral conjugated oestrogens reduce the risk of colorectal cancer by 20–30%. The effect on colorectal cancer risk of oestrogen replacement delivered by the transdermal mode has not been examined previously. We observed a protective effect for colorectal cancer that is as great, and possibly greater, in magnitude than that reported for OE replacement therapy.

Although our results for transdermal and OE were not adjusted for known important risk factors for colorectal cancer including diet and physical activity, we were able to investigate the impact of some potential covariates obtained from prospectively recorded healthcare databases. The impact of the past use of prescription NSAIDs and vitamins, the use of oral contraceptives, and the frequency of physician visits prior to index dates had a negligible effect on ORs. The minimal impact of covariates including diet and physical activity on the measures of association between HRT and colorectal cancer has been reported by other investigators (Kampman *et al*, 1997; Fernandez *et al*, 1998; Grodstein *et al*, 1998; Prihartono *et al*, 2000).

We also report results among women who had not had a sigmoidoscopy during the 3–5-year period prior to index dates. All ORs remained strongly protective for TDE and some associations became even more protective. We assumed that a sigmoidoscopy during this time period was a ‘screening’ sigmoidoscopy rather than a ‘diagnostic’ procedure. The impact of having had a sigmoidoscopy during this time could conceivably have a protective effect, if premalignant polyps had been removed (Winawer *et al*, 1993). We did not, however, have information on whether or not a polypectomy was performed, nor did we have information to indicate the reason for which a woman had had a sigmoidoscopy.

The protective effect of having had a polypectomy during a sigmoidoscopy could falsely be attributed to HRT if a woman who had used HRT had been more likely to have had this procedure performed. The association between HRT and colorectal cancer was not examined among women who had had a sigmoidoscopy 3–5 years prior to index dates, because too few women had had the procedure. However, in the majority of women who had not had a sigmoidoscopy at that time, the results for HRT remained protective. We can therefore rule out the impact of having had a polypectomy playing a role in the observed protective effect.

Women choosing to use HRT are known to be different from women choosing not to use HRT, and it had been suggested prior to the release of WHI results that some of these differences may be responsible for many of the protective effects against chronic diseases attributed to HRT (Barrett-Connor, 1991; Matthews *et al*, 1996; Brett and Madans, 1997). However, in many studies, even in well-designed studies where all known important covariates are measured prospectively (Grodstein *et al*, 1998), crude relative risks have not been very different from multivariate relative risks. It is of relevance to our results that many of the healthy lifestyle characteristics associated with HRT use are also likely to be associated with TDE use. Whether health-related differences exist between transdermal and OE users has not to our knowledge been explored. We examined the use of prescribed vitamins, NSAIDs, having had a sigmoidoscopy 3–5 years prior to the index date and frequency of physician visits among women who had been prescribed OE and TDE. The histories were very similar in these two groups. Transdermal oestrogen users were somewhat younger (mean 63.8 (s.d. 8.6) years) than OE users (mean 68.6 (s.d. 9.0) years), but all analyses were age-adjusted either by matching (conditional logistic regression) or in analyses where the matching was broken, by including age as a covariate in the model. There is no evidence to suggest that differences, if they exist, would be as extreme as the differences between HRT users and nonusers. In the analysis where women who had used OE comprised the reference group, we eliminated the important potential for bias that may exist with a comparison group of non-HRT users. The protective effect of TDE remained.

Our study is limited by the fact that we only have a small number of women with colorectal cancer who are exposed only to TDE. We do, however, have a sufficient number of exposed controls and, if TDE is truly protective, we may always have fewer exposed cases than controls. Despite a small number of exposed cases, some of our findings were significant. Nonetheless, in order to study the effect of the duration of TDE use adequately, a larger population with a longer history of TDE use must be studied.

An important strength of this study is the fact that we have highly accurate oestrogen exposure and diagnostic data, prospectively documented for all of our subjects. This avoids the potential problem of violations of temporal order, misclassification of exposure data, and selection bias due to incomplete diagnostic information, which can plague case–control studies. We also have accurate data from health service databases for all of our subjects, with regard to the dispensing of several other prescription drugs, and history of having had a sigmoidoscopy. Since 91% of the Saskatchewan population is eligible to receive provincial prescription drug benefits, our results are generalisable to the general population, with the exception of members registered with First Nations, who receive benefits under a federal program. It must therefore be cautioned that our results are not generalisable to this group.

Clinical studies have revealed metabolic differences between transdermal and OE. The induction of hepatic protein synthesis that occurs with OE administration (sex hormone-binding globulin, corticosteroid-binding globulin, thyroxine-binding globulin, transferrin, ceruloplasmin, apolipoprotein A1, rennin substrate, and various coagulation and fibrinolysis factors) is avoided with TDE (Chetkowski *et al*, 1986; Crook, 1997). The estradiol/estrone ratio produced with TDE administration is closer to premenopausal levels, than is the ratio produced with OE administration (Chetkowski *et al*, 1986; Jewelewicz, 1997; IARC Working Group on Hormonal Contraception and Post-menopausal Hormonal Therapy, 1999). In addition, with the TDE patch, the release of oestrogen is constant and the peaks and troughs characteristic of OE are avoided (Jewelewicz, 1997). The clinical relevance of these different biological properties is an area that requires study.

In summary, the reduction in colorectal cancer risk with TDE use has not previously been reported and warrants further investigation. Knowing more about the differences in health outcomes between TDE and OE could help women and their physicians individualise therapy in order to minimise the risks and maximise the benefits of treating distressing menopausal symptoms.
